# Trypsin inhibitors: promising candidate satietogenic proteins as complementary treatment for obesity and metabolic disorders?

**DOI:** 10.1080/14756366.2018.1542387

**Published:** 2019-01-07

**Authors:** Vanessa Cristina Oliveira de Lima, Grasiela Piuvezam, Bruna Leal Lima Maciel, Ana Heloneida de Araújo Morais

**Affiliations:** a Department of Biochemistry, Biosciences Center, Federal University of Rio Grande do Norte, Natal, Brazil;; b Department of Collective Health, Federal University of Rio Grande do Norte, Natal, Brazil;; c Department of Nutrition, Center for Health Sciences, Federal University of Rio Grande do Norte, Natal, Brazil

**Keywords:** Non-communicable diseases, obesity, satiety response, hormones, trypsin inhibitor

## Abstract

The increase in non-communicable chronic diseases has aroused interest in the research of adjuvants to the classic forms of treatments. Obesity and metabolic syndrome are the main targets of confrontation because they relate directly to other chronic diseases. In this context, trypsin inhibitors, molecules with wide heterologous application, appear as possibilities in the treatment of overweight and obesity due to the action on satiety related mechanisms, mainly in the modulation of satiety hormones, such as cholecystokinin. In addition, trypsin inhibitors have the ability to also act on some biochemical parameters related to these diseases, thus, emerging as potential candidates and promising molecules in the treatment of the obesity and metabolic syndrome. Thus, the present article proposes to approach, through a systematic literature review, the advantages, disadvantages and viabilities for the use of trypsin inhibitors directed to the treatment of overweight and obesity.

## Introduction

Overweight and obesity have increased the prevalence of non-communicable chronic diseases (NCDs), putting this problem at a prominent level in the world’s public health^1^^,^^2^. By 2016, more than 1.9 billion adults aged 18 years or over were overweight. Of these, more than 650 million were obese, corresponding to about 13% of the world's adult population (11% of men and 15% of women)^3^. Faced with this reality, public health policies have sought therapeutic alternatives that can help and potentiate classic interventions to change the way of life and bring a real impact in this scenario.

These classic interventions, which lead the highest degree of therapeutic recommendation, consist of limiting the energy intake of total fats and sugars, increasing consumption of fruits and vegetables, whole grains and practicing regular physical activity (60 min a day for children and 150 min throughout the week for adults)^3^. However, the obesogenic environment in which modernity has inserted us makes it difficult to adhere to this lifestyle. Despite attempts to change this epidemiological trend, the effectiveness, viability of widespread implementation, and the sustainability of such interventions need to be assessed to have a social impact^4^.

The prospection of new molecules has shown to be an interesting alternative and a way of exploring the biological variability of still unknown floras^5^. In this field, trypsin inhibitors, molecules of wide biotechnological application, have been outstanding. There is a vast literature on the biological activities of these molecules and the methods of isolation, purification and analysis on safety and their use is already well established^6^^,^^7^.

Scientific findings have shown the role of trypsin inhibitors in hormones that control energy balance and eating behaviours and have fostered research on the potential of these molecules in the treatment of chronic diseases associated to overweight and obesity^8–17^. Although the number of molecules tested in experimental models of obesity and food behaviour is still small, trypsin inhibitors have shown to be a promising alternative and capable of acting on different fronts for the treatment of these diseases.

Considering the importance of discussing the use of new adjuvants in the supplementary treatment of overweight and obesity, the present article proposes to approach through an attempt to systematically review of the advantages, disadvantages and viabilities for the use of trypsin inhibitors directed to their treatment. Thus, descriptors and key terms related to the topic were inserted in databases for the bibliographic survey. The obtained articles were analyzed for the approximation with the subject, selecting those that involved the use of trypsin inhibitors in food consumption, body weight, satiety, obesity and other related metabolic parameters.

## The impacts of obesity

NCDs are emerging as the greatest public health challenge of the early 21st century. Environmental changes and population growth, especially among the elderly population, are contributing factors to their rapid growth. Most NCDs are conditions that require expensive treatments and prolonged individual care by increasingly specialized health services. These diseases have an important impact on socioeconomic development due to productivity losses, prolonged incapacity and increased social security expenditures^1^.

Globally, at least 2.8 million people die each year as a result of being overweight or obese, and it is estimated that 35.8 million (2.3%) of disabilities adjusted per year of life have the same cause^3^. More than 80% of these deaths occur in low- and middle-income countries^3^. Epidemiological studies have begun to indicate the close relationship between diet and NCDs, diet being pointed out as one of the most difficult factors to solve.

The global shift in the food supply has increased more than ever the supply of ultra-processed, palatable, energy-rich and nutritionally poor food^18^^,^^19^. The modern environment is a potent stimulus for the reduction of levels of physical activity and the increase in caloric intake, influenced by the change in lifestyle resulting from the processes of urbanization and globalization^20^.

Thus, overweight and obesity stand out as potential causes of other NCDs. Overweight and obesity, the latter, classified as a NCD, lead to adverse metabolic effects on blood pressure, lipid profile and insulin resistance. The risks of coronary heart disease, ischemic stroke, and type 2 diabetes mellitus increase linearly with increased body mass index (BMI)^21^. The presence of overweight and/or obesity makes common the coexistence of metabolic alterations of different NCD, an increasingly common condition denominated metabolic syndrome^3^.

Up to 80% of NCD deaths can be avoided with known behavioural and pharmaceutical interventions. Several initiatives for the prevention and control of NCDs have been adopted over the past two or three decades following the World Health Assembly resolution organized by the World Health Organization. The Global Burden of Diseases has set targets for reducing NCD mortality in all nations by one third by 2030^22^.

The continued increase in obesity, hypertension, and diabetes shows the inadequacy of current strategies and raises the challenge of implementing additional strategies that may be effective in treating these diseases and have an impact on the current epidemiological picture. The search for allies that potentiate the action of traditional measures of weight reduction has directed the researches, mainly, aiming at the substitution of the drugs currently available in the market, that bring numerous side effects^23^.

Among these drugs, sibutramine, a serotonin reuptake inhibitor, was withdrawn from the European and American market due to a study that reported an increased risk of non-lethal infarction in patients with the preexisting cardiovascular disease. Orlistat, another drug popularly known in the treatment of obesity, is an inhibitor of pancreatic lipase, thus reducing digestion and intestinal absorption of fats. Flatulence, steatorrhea and fecal incontinence caused by orlistat are the main cause of discontinuation of treatment. Liraglutide is an agonist of intestinal hormones peptide-1 (GLP-1) used in the treatment of diabetes but also reduces acute food intake, subjective hunger and delays gastric emptying. However, apparently, these results are linked to its side effects such as nausea, vomiting and abdominal pain^24^. Therefore, scientific research has been looking for new drugs, especially in the field of naturally occurring molecules, whose bioactive potential is still little explored^5^^,^^25^.

## Trypsin inhibitors: concepts and mechanism of action

**Figure 1:**
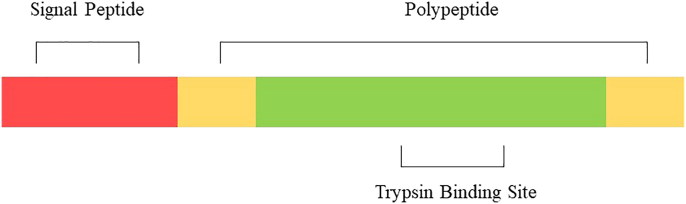
General structure of pre-protein inhibitor with highly-conserved signal peptide.

Based on their nature, inhibitors can be broadly classified into two categories: chemical inhibitors and protein inhibitors. Protease inhibitors are protein inhibitors that inhibit the action of proteases. Among the group of protein inhibitors are trypsin inhibitors (Figure 1). In the last two decades, the number of studies on plant proteases and their protein inhibitors has increased. Most studies aim to understand biochemical and physiological features that may be useful for understanding various subcellular mechanisms. These molecules can be classified by the type of protease they inhibit or by their mechanism of action.

The various classes of inhibitors were subdivided into several families based on characteristics such as homology between members, topological relationships between disulfide bridges and location of the active site. The class of inhibitors most well characterized is that of the serine proteases, widely distributed in the plant kingdom, mainly in plants of the families Brassicaceae, Cucurbitaceae, Salicaceae, Glycinea, Leguminosae, Solanaceae, Fabaceae. Currently, a number of protease inhibitors are based on the homology between molecules, meaning a significant similarity in the amino acid sequence, with a total of 83 families. The most well-characterized families of serine protease inhibitors are the Kunitz and Bowman–Birk type inhibitors^26^^,^^27^.

Kunitz-type serine protease inhibitors have molecular weights of 18–22 kDa, with a primary structure composed of about 181 amino acid residues, a binding loop that binds to trypsin, one or two polypeptide chains and a low cysteine content. The disulfide bridges are stabilized by four cysteine residues. Once these disulfide bridges are ruptured, loss of inhibitory activity is observed. Inhibitors of the Bowman–Birk type from dicots have low molecular mass, ranging from 8 to 10 kDa, a high content of cysteine and two reactive site loops per molecule, each with an affinity for a range of serine proteases such as trypsin, chymotrypsin, elastase, and kallikrein. Already inhibitors of the Bowman–Birk type of monocots are divided into two subclasses with different molecular weights: ∼8 kDa and ∼16 kDa. Unlike Kunitz type inhibitors, which are encoded by a single gene, the Bowman–Birk family is encoded by at least three genes for the synthesis of their isoforms^6^^,^^28^.

**Figure 2:**
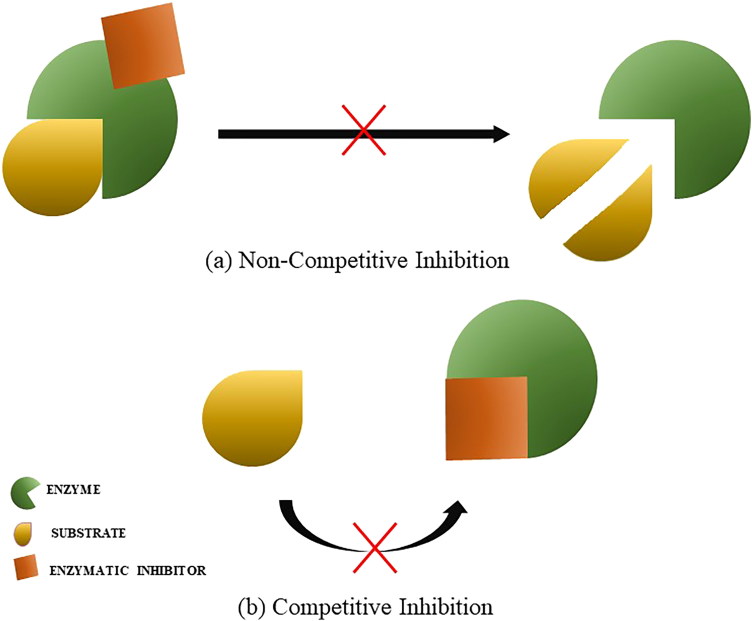
Mechanisms of enzymatic inhibition. (a) non-competitive inhibition. (b) competitive inhibition.

These enzyme inhibitors bind to their substrates by different mechanisms, with some exceptions. When this bond forms a complex they are called irreversible. When not, it can be classified according to the binding site in competitive and non-competitive inhibition. The vast majority of protease inhibitors act on their targets by the competitive inhibition mechanism. Despite divergent targets and different mechanisms of inhibition, most protease inhibitors bind a critical portion of the inhibitor in the active site in a substrate-like manner^29^ (Figure 2).

Due to the property of inhibiting enzymes by the most diverse mechanisms, one of the well-studied applications of plant protease inhibitors is their use in the control of pests and pathogens, especially in the field of trypsin inhibitors^30^. In the field of nutrition, trypsin inhibitors have long been considered as antinutritional factors that compromise the nutritional quality of foods. The idea that trypsin inhibitors would interfere with the digestive processes of animals is not recent and arose from the observation that many trypsin inhibitors were able to protect plants from insect digestive enzymes^31^. The reduction of animal weight in experimental models caused by trypsin inhibitors was associated with their deleterious effects on the nutritional status of the animals^32^^,^^33^.

## Trypsin inhibitors: heterologous applications for health

Trypsin inhibitors have great potential for clinical application in addition to their role in plant defence^34^. Some trypsin inhibitors found in legumes have important anticarcinogenic and radioprotective activity, reducing the incidence of cancers of the colon, prostate, breast and skin in the populations that consume them^35^. This effect is possibly the result of the inhibition of proteolysis caused by the transformed cells since they require the proteases for the events of metastasis, angiogenesis, tumour growth and proliferation^7^^,^^36^.

Trypsin inhibitors also regulate the activity of some serine proteases such as neutrophil elastase. These enzymes are present in inflammatory cells or neutrophils, and the uncontrolled release of these molecules causes severe tissue damage^37^^,^^38^. Because of these properties, they may act in the treatment of diseases involving elastase release as gastric ulcer and pulmonary diseases such as emphysema^39^^,^^40^.

Some cardiovascular conditions, such as myocardial infarction, venous thromboembolism, stroke, and sepsis, are linked to inappropriate serine protease activity involved in the blood coagulation cascade^41^. Some trypsin inhibitors of the Kunitz family and the corn trypsin inhibitor are well characterized by the blocking activity of proteases involved in platelet aggregation, blood coagulation, fibrinolysis and coagulation^42^^,^^43^.

Reports of the relationship between inhibitors and body weight change are not recent. Initially, some studies reported the relationship between intake or supplementation of inhibitors and reduction of body weight in experiments with animals, indicating this weight loss as a deleterious factor^44^^,^^45^. However, it has been observed that this relationship between the administration of trypsin inhibitors and the weight changes could be a reflection of other physiological effects of these molecules^46^. Thus, research was directed to the application of these inhibitors in experimental models that aim to evaluate their performance in specific nutritional and metabolic situations, such as NCDs, many of these approaches related to the action of these proteins on satiety hormones.

## Trypsin inhibitors in the regulation of appetite and energy balance involving satiety hormones

The brain-intestine axis is an interdependent system, which affects neural function and controls our eating behaviour through biochemical signalling between the endocrine and nervous system, involving hormonal peptides in the gastrointestinal tract. The two main families of gastrointestinal hormones are those of appetite stimulants, being represented by ghrelin, a peptide that increases the appetite and the feelings of hunger; (PYY3-36), cholecystokinin (CCK) and leptin, which signal the reduction of hunger in the brain and promote cessation of the meal. Insulin, a pancreatic hormone, also plays an important role in human metabolism and in eating behaviour^47^.

Metabolic syndrome and obesity are NCDs generally associated with increased energy intake in relation to calorie requirements and delayed satiety. Since alterations in gastrointestinal motor functions in obesity may present useful targets for preventing and treating metabolic disorders, the management of gastric emptying by hormones, such as CCK, may be a good strategy to reduce the impacts associated with these metabolic diseases^48^.

The relationship of CCK to satiety is well established. In animal model, CCK induce reducing the intake of food in the stomach and intestine, and in the central nervous system, leading to the development of a "satiety behaviour". CCK interacts with other hormones to control food intake and the satiety induced by this hormone is enhanced when combined with other anorectic signals. CCK was the first gastrointestinal peptide involved in the control of food intake and, later called the "satiety signal"^49^^,^^50^.

This hormone is produced by the I cells of the small intestine and jejunum, and some regions of the nervous system, in response to the presence of products of the digestive process, such as partially digested proteins and lipids^51^. The physiological effects of this hormone are: to stimulate exocrine pancreatic secretion and gallbladder contraction, which are critical for the digestion of these nutrients; to regulate gastric emptying and intestinal transit to improve nutrient absorption, preventing overload of the absorptive process in the intestine; to stimulate satiety centers in the brain by acting on receptors expressed in vagal afferent neurons^52^.

The elucidation of the mechanisms underlying the central nervous system responses to circulating CCK has also been a subject of intense interest. Studies over many years have contributed to the idea that CCK released from the small intestine acts directly on vagal afferent neurons that terminate in the nucleus of the solitary tract and activate upward pathways that control food behaviour^53^^,^^54^. However, CCK acts on CCK1 receptors to stimulate expression of the CART (Cocaine and Amphetamine-Regulated Transcript) and Y2 receptor gene, both linked to inhibition of food intake, while suppressing the expression of MCH (Melanin-Concentrating Hormone) and MCH-1 and cannabinoid (CB-1) receptors, which are linked to stimulation of food intake^55^.

In addition, there is an integrative response due to co-expression of other gastrointestinal hormones, acting collaboratively in the satiety response. Some nutrients, such as lipids, act by stimulating the joint secretion of CCK, PYY, and GLP-1 that, in addition to other functions, initiate the process of reducing meal and gastric motility. This is due to the fact that G protein-bound receptors (GPRs), such as GPR40 and GPR120, are expressed in enteroendocrine cells secreting PYY and GLP-1, in addition to CCK. Leptin, a hormone that is related to body adiposity and that acts on body energy balance, can also act synergistically with CCK. The long form of the leptin receptor is co-expressed with the CCKR1 receptors, generating a cooperative effect between leptin and CCK^56^.

Insulin is also related to body adiposity. Like leptin, the insulin produced is transported to the brain, and its direct administration in that tissue reduces food intake^57^. Insulin, similar to leptin, also has a synergistic effect on CCK. CCK is capable of increasing insulin transport to the central nervous system, an effect probably mediated by CCKR1 receptors, expressed on the membrane of the blood-brain barrier. This mechanism is still not well understood, but it is possible that CCK also reduces insulin degradation. Even though it does not cross the blood-brain barrier, CCK collaborates in this transport of catabolic signals^58^.

Restricting energy intake seems to be the most effective way to lose weight and improve glucose control in individuals with obesity or diabetes. However, attempts to sustain significant weight loss through lifestyle intervention often fail. The results obtained through anorexigenic drugs are an indirect consequence of their adverse effects such as nausea, pain, anxiety, ataxia, sedation or hyperactivity^59^, besides the low adhesion rates that these treatments generate^60^. As inhibition of intake *per se* cannot be taken as a reliable indicator of satiety, we have sought adjuvants that can act more globally, not only in satiety but also in eating behaviour. Considering the physiological effects caused by satiety hormones, they have been the object of research and studies that relate them to eating behaviour and weight changes^49^^,^^61^.

**Figure 3:**
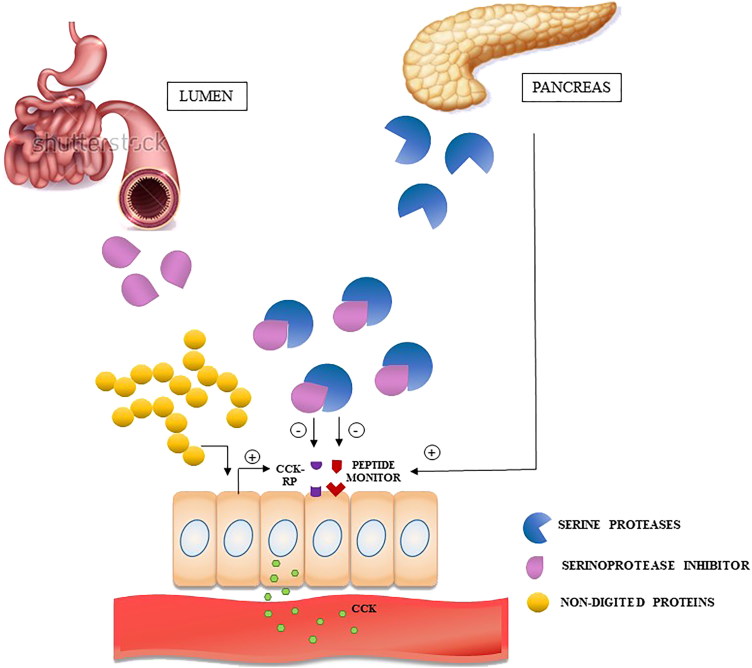
Intestinal mechanism of CCK release induced by the presence of trypsin inhibitors.

One way to improve dietary adherence rates in clinical practice may be to increase satiety through the use of trypsin inhibitors. The probable mechanism for inducing satiety by these trypsin inhibitors may be related to the common ability to increase serum concentrations of CCK^11–15^. Inhibition of digestive enzymes caused by the administration of these inhibitors may lead to the accumulation of undigested proteins in the intestine, culminating in the greater release of CCK (Figure 3), which, in addition to act delaying gastric emptying, modulates satiety responses in the neural energy regulation^62–64^.

In 1983, the study by McLaughlin et al. related the administration of soybean trypsin inhibitor (SBTI) with the release of CCK and its role in controlling food intake. Since then, the literature on the relationship of these inhibitors and the release of CCK has been highlighting, from the investigation of this mechanism in inhibitors extracted from other plant sources^46^.

Despite the fact that the first signs of the relationship between trypsin inhibitors and CCK, the subsequent studies with the soybean inhibitor did not present promising results. *Wistar* rats fed SBTI presented a reduction in weight gain. However, these animals also showed a reduction in protein digestibility when compared to the group treated with casein. Thus, being the effect conditioned by a deleterious action of SBTI on the ability of animals to obtain dietary protein^65^.

β-conglycinin, a protein representing the largest fraction of soy protein, was able to reduce gastric emptying and food intake in *Sprague–Dawley* rats and also increase CCK secretion. Unlike the previous findings, the study found that the mechanism of action of β-conglycinin was not related to the reduction of trypsin activity, but to its binding to intestinal epithelial cells^8–10^, more specifically, the β-conglycinin β51-63 peptide, rich in arginine^66^. Other studies have sought to clarify the mechanisms adjacent to CCK release further^67–69^.

Other plant sources have shown to be more promising in obtaining trypsin inhibitors with satiety potential. The potato contains high concentrations of these molecules, accounting for 20–50% of its soluble proteins. A peptide with potent antimicrobial activity and sequence homology with a protease inhibitor that is produced by potato tubers after a fungal attack has been identified. Interestingly, the N-terminal sequence of that peptide was identical to the corresponding sequence of the proteinase inhibitor member of the Kunitz superfamily^70^. The major studies on these inhibitors and satiety started with the isolation of the potato trypsin inhibitor (POTII). POTII administration in a meal to lean adult subjects led to a 17.5% reduction in caloric intake at the next meal compared with the addition of other dietary proteins^71^.

With the isolation of this inhibitor, its effect when concentrated was analyzed and at the end of fourteen days of oral ingestion of this concentrate of potato inhibitor (PPCI), the reduction of body weight gain was observed by reducing food consumption in healthy *Wistar* rats when compared to a control protein (casein), without causing deleterious effects. This adaptation of body weight appeared to be time dependent as it took at least 6 days for the inhibitor to induce significant changes in body weight gain. According to Komarnytsky et al. ^11^ , this greater satiety activity could be attributed to the greater total antitryptic activity of the concentrate, due to the possible presence of other inhibitory molecules.

Nakajima et al. ^12^ evaluated the ability of a potato extract containing trypsin inhibitor, Potein, to suppress food intake in *Sprague–Dawley* rats and CCK secretion in STC-1 cell culture compared to the inhibitor of soybean trypsin (SBTI). Increasing the dose of Potein used led to a reduction in food intake in a dose-dependent manner 3–6 h after its administration. Regarding the release of CCK by enteroendocrine cells, Potein also presented a dose-dependent relationship, even presenting lower antitryptic activity than SBTI, which did not stimulate the secretion of CCK in STC-1^12^. Other studies have sought to further clarify this mechanisms adjacent to the release of CCK

The promising effects led to the patenting of the potato inhibitor, later marketed as Potein^®^. Potein^®^ is a potato juice preparation produced during the processing of the starch containing protein fractions with antitryptic activity. Chen et al. ^13^ also evaluated the performance of Potein^®^ administered before a meal in *Sprague–Dawley* rats. Food intake between 1 h and 3 h after orogastric administration of the extract (1.5 g/kg) tended to be lower, with a significant effect 6 h after administration. When, prior to orogastric administration of Potein^®^, devazepide, a CCK receptor antagonist, was administered intraperitoneally, no reduction in dietary intake was observed. When administered directly into the animals' duodenum, Potein^®^ was able to increase CCK concentrations by 25% over control^13^.

Another promising source of an inhibitor with satietogenic activity are the seeds of the tamarind fruit. The trypsin inhibitor isolated from *Tamarindus indica* L. (TTI) was able to reduce the food intake of eutrophic *Wistar* rats by about 47%, leading to a reduction in weight gain of approximately 70% compared to the control group when compared to a standard protein or SBTI. *In vivo* protein digestibility tests showed that this reduction was not associated with deleterious effects of the inhibitor, but rather with the development of some satiety mechanism. This mechanism was associated with CCK activity whose serum levels were significantly higher in animals that received a diet containing TTI, but without significant difference between the doses administered. Histological analyses of liver and pancreas did not demonstrate pathological changes in the group supplemented with the trypsin inhibitor^14^.

Subsequently, the same tamarind seed inhibitor was evaluated in *Wistar* rats with diet-induced obesity and given by gavage over a period of 10 days. No difference in weight gain was observed between TTI and obese rats, both on diet-induced obesity. However, the rats treated with the inhibitor showed a consumption reduction significantly higher, indicating a possible satiety effect caused by TTI^16^.

In a study similar to that of Carvalho et al.^16^, Costa et al.^72^ also evaluated the effect of TTI on an experimental model of obesity. It was observed that TTI was able to reduce food intake in animals submitted to an unbalanced diet, causing discrete improvements in zoometric indexes, however, without altering their nutritional status. In evaluating the expression of CCK-related genes, such as the *CCK1R* and *CCK2R* receptor genes, and the convertase protein that participates in the post-translational modifications of CCK, it was observed that obese animals treated with standard diet significantly reduced the relative expression of mRNA *CCK1R*. However, TTI was also able to decrease the relative expression of this gene in obese animals when compared to those without treatment. This may be considered a positive effect since it has also been observed in obese animals receiving a standard diet, which may be related to the sensitivity to the effects of CCK^72^.

A trypsin inhibitor isolated from peanut *paçoca* (typical candy of the Brazilian cuisine) also had positive effects on food consumption control, although it was isolated from a high-calorie food. In a study prior to the use of trypsin inhibitor isolated from peanut *paçoca*, the authors used peanut *paçoca* in diet supplementation of eutrophic Swiss mice and demonstrated a decrease in body weight gain and food intake when administered for 10 days. However, this effect could not be attributed to the inhibitor, considering the diversity of the nutritional composition of peanut *paçoca*^73^.

In a later study, when isolated from peanut *paçoca*, this trypsin inhibitor was tested in eutrophic *Wistar* rats using the same experimental model used in mice, showing similar results and additionally the animals treated with the inhibitor showed an increase in serum concentrations of CCK in a dose-dependent manner. Thus, the reduction of weight gain was related to satiety, since the animals showed no evidence of impaired nutritional status^15^.

Considering the studies presented and aiming to summarize the information related to the main information and gaps perceived in this review of the literature, 13 articles on trypsin inhibitors and their relationship with satiety and food consumption were gathered in Table 1.

**Table 1.** Results of the search in databases on trypsin inhibitors, satiety, food consumption and obesity.

Trypsin inhibitor	Study	Assessed parameters	Experimental model	Experimental design	Results
Synthetic Trypsin Inhibitor (N, N-dimethyl-carbamoyl 4- (4-guanidino-benzyloxy) -phenyl acetate methane sulfate)	McLaughlin et al., 1983.[46]	Food behaviour, food consumption, and weight gain.	Adult lean male (*n* = 5) and female (*n* = 12) *Zucker* rats, and adult obese male (*n* = 5) *Zucker* and female (*n* = 12) rats. Obesity was induced by a genetic modification in the *fa/Nofa* gene	In the experiment on feeding behaviour, obese and lean male rats, pre-trained to tighten a feed rod after gavage with water, received the synthetic trypsin inhibitor for 4 weeks. In the experiment of food consumption and weight gain, obese and lean females were evaluated for one week regarding consumption and daily weight gain.	Reduction of intake and mean meal size in a dose-dependent manner in obese and lean rats, but with a greater response in obese rats. Reduction of body weight in rats with obesity, but not lean. These results were attributed to the effect of trypsin inhibitors on CCK.
Soybean (SBTI/ β-conglycinin)	Nishi et al., 2003.[9]	Food consumption, gastric emptying, secretion and plasma concentrations of CCK	Adult lean male *Sprague–Dawley* rats (*n* = 8; *n* = 5–6); culture of small intestine cells of *Sprague–Dawley* rats.	In the *in vivo* experiments, a duodenal cannula was inserted for β-conglycinin administration. Food intake was then measured 30 or 60 minutes after infusion. Plasma concentrations of CCK were evaluated 45 minutes after β-conglycinin administration. For gastric emptying, in addition to the duodenal cannula, in which β-conglycinin was infused, a gastric cannula was used for infusion and collection of gastric contents. For the *in vitro* experiments, a culture of rat intestinal mucosa cells was used to evaluate the release of CCK in the presence of β-conglycinin, and the direct binding to these cells.	Suppression of food intake by β-conglycinin in a CCK-dependent manner and inhibition of gastric emptying. Β-conglycinin binds to rat intestinal membrane components and stimulates the release of CCK directly into cells.
	Nishi et al., 2003.[9]	Food consumption, plasma concentrations of CCK and interaction of the molecule with the brush border membrane.	Adult lean male *Sprague–Dawley* rats (*n* = 9; *n* = 8; *n* = 10); culture of proximal small bowel cells of *Sprague–Dawley* rats.	In the *in vivo* experiments, a duodenal cannula was inserted for administration of peptides derived from β-conglycinin degradation. After infusion, food consumption was measured 60 minutes after the offer of 15g of the standard diet. Plasma CCK concentrations were assessed 45 minutes after β-conglycinin peptide administration. For the *in vitro* experiments, a mouse intestinal mucosa cell culture was used to evaluate the binding of β-conglycinin peptides to the brush border membrane of these cells.	The administration of β-conglycinin peptides suppressed food intake in a CCK-dependent manner. The β 51-63 peptide showed the most robust binding to the brush border membrane. The 3mmol / L concentration of β 51-63 presented more visible results regarding the suppression of food intake and CCK release.
	Sufian et al., 2011.[10]	Food consumption, secretion of CCK acute and chronic	Adult lean male *Sprague–Dawley* rats (*n* = 5–6); culture of STC-1 cells	The secretion of CCK in STC-1 cells was evaluated from the incubation of different β-conglycinin hydrolyzed for 60 minutes with food-processing proteases thermolysin (BconT), bromelain (BconB), chymotrypsin, protease S, and protease M. The β-conglycinin peptides which showed CCK release activity was administered to rats via gavage after a 24h fast. After 30 minutes, 25g of diet was offered, and the food consumption evaluated for 60 minutes. One of the peptides was tested for a chronic period (14 days) in two concentrations for two daily periods.	Incubation with different β-conglycinin hydrolysates led to CCK secretion, with better results for the BconB peptide. In the acute suppression of appetite, two peptides obtained similar results, and a third presented no consequence. The BconB peptide was used in the evaluation of chronic consumption, showing an overall reduction in intake at both concentrations, with more potent effects at the lowest dose tested.
Potato (POT II/PPIC/Potain^®^)	Hill et al., 1990.[71]	Food consumption, motivation to feed and food preferences	Lean men (*n* = 3) and women (*n* = 8).	The subjects answered a questionnaire of motivation to eat and food preference. Then a soup dish with or without POTII was offered. Immediately after the soup, or after five minutes of waiting, individuals assessed their eating motivation and food preferences and were then presented with the test meal. Consumption was evaluated. Other evaluations of motivation to eat and food preference. The rest of the day, the food diary was full.	The addition of POTII to the soup significantly reduced energy consumption by an additional 17.5%. The evaluation of the motivation to eat and food preferences did not predict the reduction in energy intake by the proteinase inhibitor. This result was attributed to the greater release of CCK by POTII, suggesting a possible therapeutic potential.
	Komarnytsky et al., 2011.[11]	Gastric emptying, food consumption, proteolytic activity, plasma CCK, toxicity and *CCK* gene expression	Adult lean male *Wistar* rats (*n* = 6–8); culture of STC-1 cells; culture of *Salmonella*	Food intake was evaluated after acute intake of PPIC by gavage during a 24-hour period. Food intake and weight were evaluated during administration for 10 days of a dose of PPIC, as well as dosages of CCK before and after gavage. Gastric emptying and enzymatic activity were evaluated after a single dose of PPIC. Toxicity for 14 days was evaluated by hematological, biochemical and pathological parameters. The mutagenic potential of PPIC was evaluated in the culture of bactéria. *CCK* expression was evaluated in STC-1 cells.	Reduction in immediate food intake. Repeated oral ingestion of PPIC reduced weight gain in male rats and significantly increased plasma CCK concentrations through a trypsin-dependent mechanism. PPIC also retarded gastric emptying and decreased proteolytic activity in the duodenum of healthy rats. CCK mRNA expression of duodenal mucosa increased in response to PPIC administration, but did not alter expression or secretion of CCK in STC-1 cells. PPIC appeared to be safe and nontoxic in these studies.
	Nakajima et al., 2011.[12]	Food consumption and secretion of CCK	Adult lean male *Sprague–Dawley* rats (*n* = 16; *n* = 11); culture of STC-1 cells.	Potein, SBTI and water were administered by gavage. In a first experiment, inhibitors were administered 40 minutes before the meal. In a second experiment, different doses of Potein were administered. In a third experiment, a solution with composition similar to Potein was offered. In the fourth experiment, Potein, SBTI and water were offered 2 hours before the meal. In all experiments, food consumption was evaluated at 1, 2, 3 and 6 hours after the onset of feeding. Secretion of CCK against Potein and SBTI were examined in STC-1 cells after enzymatic digestion.	Suppression of food intake for 1h to 6h in a dose-dependent manner and direct induction of CCK secretion in STC-1 cells. Digestion of Potein with protease did not attenuate its ability to induce CCK secretion in STC-1 cells. These results suggested that oral administration of Potein in rats suppressed food intake via CCK secretion induced both by stimulation in CCK-producing cells and by inhibition of luminal trypsin.
	Chen et al., 2012.[13]	Food consumption, plasma CCK and CCK secretion.	Adult lean male *Sprague–Dawley* rats (*n* = 12; *n* = 07; *n* = 20); culture of STC-1 cells.	Food intake was evaluated 1h, 2, 3h and 6h after administration of two different concentrations of Potein^®^, or after administration of Potein^®^ and other protein sources; or after administration of Potein^®^ and a CCKA receptor inhibitor. The release of CCK was evaluated *in situ* using a catheter inserted into the portal vein after infusion of Potein^®^ directly into the duodenum. Secretion of CCK against Potein^®^ was examined in cell culture.	Significant reduction of food intake 6 h after the administration with increase in plasma CCK. Increased secretion of CCK in STC-1 cells relative to control.
Peanut (AHTI)	Serquiz et al., 2016.[15]	Food consumption, weight, biochemical parameters and histology of the pancreas.	Adult lean male *Swiss* males mice (*n* = 8).	The animals were fed a diet enriched with peanut *paçoca* for 14 weeks. During this period, weight and food consumption were observed. After follow-up, blood glucose, lipid profile, liver transaminases and histology of the pancreas were evaluated.	Weight loss and lower average food consumption at the end of follow-up, with no signs of pancreatic toxicity. The hypercaloric diet composed of *paçoca* presented antitryptic activity.
	Serquiz et al., 2016.[15]	Food intake, protein digestibility, plasma CCK and biochemical parameters.	Adult lean male *Wistar* rats (*n* = 05).	The animals were fed for 11 days with standard diet and administration of AHTI via gavage. During this period, food consumption was assessed and feces and urine were collected. The protein digestibility was evaluated and at the end of the follow-up, the blood was collected for glucose dosage, lipid profile, and liver transaminases. In a second experiment, the animals received AHTI via gavage for 11 days. Food consumption was evaluated 1h, 2h and 18h after gavage. At the end of the experiment, blood was collected for CCK dosing.	Reduction of consumption resulting from the reduction of body weight, without alteration of protein digestibility. Reduction in fasting blood glucose. AHTI showed antitryptic activity and led to the dose-dependent increase in plasma CCK.
Tamarind (TTI/TTIp)	Ribeiro et al., 2015.[14]	Food consumption, weight, biochemical and histomorphological parameters of organs.	Adult lean male *Wistar* rats (*n* = 06).	The animals were fed for 11 days with standard diet and administration of TTI via gavage. During this period, food consumption was assessed and feces and urine were collected. The protein digestibility was evaluated and at the end of the follow-up, the blood was collected for glucose dosage, lipid profile, and liver transaminases. In a second experiment, the animals received AHT gavage for 11 days. Food consumption was evaluated 1h, 2h and 16h after gavage. At the end of the experiment, blood was collected for C-reactive protein and CCK plasma dosage.	Reduction of food intake and weight gain without alteration of protein digestibility and organ histology. TTI showed antitryptic activity and led to the dose-dependent increase in plasma CCK.
	Carvalho et al., 2016.[16]	Food consumption, weight, biochemical and inflammatory parameters.	Adult male *Wistar* rats (*n* = 05) with obesity and metabolic syndrome. Obesity was induced by high glycemic index and glycemic load diet.	A diet with high glicemic index and load was offered for 17 weeks. During the last ten days of this period, TTI was administered daily by gavage, and food consumption and weight were evaluated. After follow-up, the blood was collected for analysis of glycemia, lipid profile, liver enzymes and inflammatory cytokines.	Reduced food intake without changes in weight gain. Reduction of plasma concentrations of tumour necrosis factor alpha (TNF-α).
	Medeiros et al., 2018.[17]	Concentrations of plasma CCK and leptin.	Adult obese (*n* = 05) and lean (*n* = 05) male *Wistar* rats. Obesity was induced by high glycemic index and load diet.	A diet with high glicemic index and load was offered for 17 weeks. During the last ten days of this period, TTIp was administered daily by gavage, and food consumption and weight were evaluated. After follow-up, blood was collected for leptin and CCK plasma.	Reduction of plasma leptin concentrations. There was no influence on plasma CCK concentrations.
	Costa et al., 2018.[72]	Food consumption, weight, expression of genes related to CCK and leptin and their respective plasma concentrations.	Adult obese (*n* = 05) and lean (*n* = 05) male *Wistar* rats. Obesity was induced by high glycemic index and load diet.	A diet with high glicemic index and load was offered for 17 weeks. During the last ten days of this period, TTI was administered daily by gavage, and food consumption and weight were evaluated. After follow-up, the blood was collected using leptin and CCK plasma concentrations. Subcutaneous adipose tissue was used to evaluate gene expression.	Increased expression of the *CCK1R* gene without changes in plasma CCK concentrations, *CCK* gene expression, *CCK2R* receptor and *PSCK1* processing enzyme. Reduction of plasma concentrations of leptin without altering the expression of its respective gene.

In view of the above, despite the promising effects, the use of CCK secretagogues still faces some challenges. The studies do not show homogeneity regarding the animal model used, nor time of the experiments. Another important issue is that the follow-up time of the studies is relatively long in relation to the animal's life span, between 10 and 20 weeks, including induction of the disease, in the case of studies in obese animals, which eventually involves relative biases to the natural aging process of the animals^74^. Also, there is no standard for the obesity induction protocol.

Relative to the molecule, it is known that CCK is a molecule associated with satiety in the short term. The response to CCK can also be a confounding factor. Some diet-induced obesity, such as that induced by a hyperlipidic diet, alter sensitivity to CCK, whereas, in some animal models, the low sensitivity to CCK is a condition that preceds obesity^75^. Finally, the mechanism of satiety by the action of the inhibitors needs further clarification, since it is not always linked to its antitryptic activity^12^. The studies showed another potential that may generate other research questions and may also be related to satiety induction mechanisms, a result common to all 13 studies analyzed. Many studies involving inhibitors with claims of satiety have stagnated in terms of recent publications, which limits the deepening of their molecular mechanisms. Trypsin inhibitors, however, continue to be studied, which in the future will make it possible to uncover and fill information gaps presented in the published studies.

## Trypsin inhibitors in the treatment of chronic diseases: challenges and future prospects

Obesity and the metabolic syndrome are conditions that lead to an extensive metabolic dysregulation. Excessive accumulation of adipose tissue, especially in the visceral region, increases the release of free fatty acids and the secretion of adipokines. Consequently, the body develops a chronic inflammatory state, which together with the excess of adipose tissue leads to a state of insulin resistance, constant oxidative stress, and altered immune system. Because of these characteristics, the molecules involved in the treatment of these diseases need to have a more comprehensive action to promote a sensible benefit in the state of health^76^. Because they possess an extensive number of heterologous biological activities catalogued, some trypsin inhibitors are capable of acting on several fronts in the treatment of complex diseases as discussed herein. The trypsin inhibitor isolated from peanut *paçoca* (AHTI) was able to reduce fasting glycemia in *Wistar* rats fed standard ration, considered nutritionally adequate for these animals^15^. Other trypsin inhibitors already described in this study, when evaluated in diets supplemented or administered by gavage to healthy rats, did not induce any alteration in plasma glucose, total cholesterol, triglycerides and liver transaminases in relation to their respective control groups^11–13^. In addition to the performance on fasting glycemia, AHTI was able to reduce feed consumption and weight gain of the animals. In addition, the inhibitor, even after isolated from a heat process, showed that it was a stable and resistant molecule^15^.

The trypsin inhibitor isolated from bitter gourd (*Momordica charantia* Linn), a widely consumed vegetable in subtropical regions, showed properties of control of hyperglycemia. Bitter gourd was already used in folk medicine for this purpose, but this fact was proven by the interaction of the inhibitor with the insulin receptor. Experiments and an animal model showed that intraperitoneal administration of the inhibitor mcIRBP in healthy rats and with type I diabetes was able to reduce fasting glucose concentrations in the two groups^77^^,^^78^.

Soy proteins have been shown to have several functional properties, mainly related to cardiovascular parameters, altered in overweight and obesity conditions, and in chronic diseases due to these conditions^79^. Studies related to the activity of these proteins have attributed to β-conglycinin a large part of these functional properties. Gene expression in the mouse liver is altered after consumption of β-conglycinin, accompanied by a decrease in blood glucose and insulin levels. The most significant increases were in the expression of the fibroblast growth factor 21 (*FGF21*) gene and in hepatic and circulating levels of *FGF21*, which resulted in a reduction in body weight gain in response to hyperlipid diet intake. More surprisingly, single ingestion of β-conglycinin after overnight fasting was sufficient to induce an increase in the expression of the *FGF21* gene in the liver and the circulating levels of postprandial *FGF21* This change triggers a concomitant increase in lipolytic gene expression in epididymal white adipose tissue, thus exerting beneficial effects on health^80^.

In addition to acting on specific deposits of body fat, β-conglycinin also demonstrates activity on the hepatic deposition of lipids. OLETF rats fed a sucrose-inducing obesity diet, in which β-conglycinin partially replaced the protein source, showed significant differences in serum and hepatic lipid concentrations compared to their controls. Total serum cholesterol and serum phospholipid concentrations were lower in β-conglycinin-fed mice than in controls, as was serum CRP. Also, lower levels of hepatic triglycerides, cholesterol, and phospholipids were observed in the β-conglycinin group. The activity of the hepatic enzyme fatty acid synthase (FAS) was also reduced in the group supplemented with β-conglycinin, just as the enzyme gene was less expressed in that group. Thus, the dietary intake of β-conglycinin has been shown to reverse some of the metabolic abnormalities related to obesity observed in OLETF mice. In particular, the hypolipogenic function of β-conglycinin in the liver may provide a mechanistic basis for the observed effects^81^.

These results and other indications reinforce that β-conglycine may have a potential effect on nonalcoholic hepatic steatosis, a common complication in obesity^82^. In the study by Li et al.^83^, supplementation of male mice submitted to a hyperlipid diet induced a reduction in weight gain. Besides, β-conglycinin suppressed the hepatic expression of Pparγ2, the regulatory element binding protein of esterol-1c (SREBP-1c) and its target genes. In addition to these effects, β-conglycinin also induced the reduction in serum leptin and insulin concentrations and reduction of subcutaneous adipose tissue^83^.

Tamarind trypsin inhibitor when administered by gavage in *Wistar* rats with obesity and metabolic syndrome induced by diet, besides causing the reduction of the food consumption in these animals, also reduced serum TNF-alpha, regardless of the weight loss^16^. TNF-alpha is recognized as a central inflammatory cytokine in the inflammatory response of the metabolic syndrome and obesity^84^.

Subsequent studies involving the trypsin inhibitor of tamarind seeds, isolated^72^ and purified^17^ these proteins administering them by gavage in *Wistar* rats with diet-induced obesity, evaluating its action on CCK and leptin. At both stages of purification, in both studies, the inhibitor was able to reduce serum leptin concentrations five-fold compared to the untreated obesity group.

Purification of an inhibitor extracted from natural sources is an important step since its activity can be altered when comparing a rich to a purified fraction. TTI has already been purified and part of its sequence has been characterized. In the process, two fractions, Fr1 and Fr2, were obtained, with molecular masses of 19.59 kDa and 19.57 kDa, 54 and 53 amino acids, respectively, both with the same sequence, which lead to the conclusion they were the same molecule. Purified TTI, called TTIp, is heat resistant, presenting 100% antitryptic activity at the maximum temperature of 80° C, presenting IC50 of 2.7 × 10^−10 mol/l^ and *K_i_* of 2.9 × 10^−11 mol/l^. The primary sequences obtained showed high homology with the Kunitz-type soybean trypsin inhibitor, classifying it as belonging to the Kunitz-type family^17^.

Unlike what was observed in the study by Ribeiro et al.^14^, , in these studies there was no influence on CCK concentrations in obese animals. It was assumed that the satiety effect of the tamarind inhibitor has no connection neither with the antitryptic activity nor with the modulation of CCK concentrations^14^^,^^17^^,^^72^.

As already described, a series of hormones with satiety activity contribute to energy balance and food consumption regulation. These studies have pointed out that trypsin inhibitors may not have exclusive action on CCK but also on leptin^17^^,^^72^.

Circulating leptin increases the sensitivity of vagal afferent neurons to CCK, promotes the expression of vagal afferent neurons of receptors and neuropeptides associated with inhibition of food intake, and inhibits the expression of receptors and neuropeptides associated with stimulation of food intake. The long form of the leptin receptor is co-expressed with CCK1R in a subset of vagal afferent neurons. Leptin alone rapidly increases the discharge of these neurons and increases cytosolic calcium^85^^,^^86^.

Many of the neurons expressing CCK1R also express the OB-Rb receptors of leptin. The expression of these receptors in the nodose ganglia is not homogenous. CCK alone stimulates only a third of these neurons. Of the remaining two thirds, one responds exclusively to leptin, and the other to both hormones. Of the neurons that respond exclusively to CCK, two-thirds respond only to high concentrations of the hormone^87^. Thus, it is possible that leptin also stimulates CCK1R receptors, as observed in the study by Lima et al., in which a high glycemic index diet increased leptin *Ob* and *CCK1R* gene expression^88^. The reduction of leptin concentrations, known to be high in individuals with obesity, may be essential for the improvement of sensitivity to this hormone, as well as CCK since they share synergistic actions in the sign of satiety^89^.

The discovery of molecules that have potential to treat diseases of great social relevance as chronic diseases, which are among the leading causes of death in the world, is a long and challenging journey. Working with animal models is imperative, especially with a species that shares characteristics of human obesity and its comorbidities. Although there is a clear and well-documented genetic component for the trend toward obesity, most cases of human obesity are still considered a result of the integrated activity of numerous genes and their relationship to the environment. Trying to mimic an obesogenic environment in an animal model has been a necessity^90^.

Because it is one of the main factors for the development of obesity and metabolic syndrome, the development of diets that reproduce pathophysiological findings in humans has been sought. The hyperlipidic diets, containing about 40–60% of the energy coming from this nutrient from food sources mostly composed of saturated fats, are able to induce, in addition to weight gain, insulin resistance. On the other hand, hyperglycemic diets, based on simple carbohydrates (about 60% of total energy), induce weight gain, increased body fat deposition, triglycerides, hyperglycemia, inflammation, and hyperleptinemia^88^^,^^91–93^. Performance responses may also vary due to the nutritional status of the animal model and species. Moreover, it is known that in obesity different results may be observed when applying treatments as opposed to what is observed in situations of eutrophy^94^^,^^95^.

Studies involving trypsin inhibitors and obesity mostly direct administration of the inhibitors to the gastrointestinal tract. The submission of these molecules to the digestive process can generate transformations in their structures that are difficult to predict *in vitro* or *in silico* models. In this way, investing in new technologies that allow the encapsulation of these molecules in matrices that guarantee the delivery of the intact product is now being used. Encapsulation promotes not only viability but, more importantly, it also protects functionality and can facilitate targeted release in specific parts of the body. Different encapsulation approaches qualify for the protection of bioactive components^96^.

Chanphai and Tajmir-Riahi^97^, encapsulated a soybean trypsin inhibitor with chitosan. Positives changes after encapsulation were observed by UV/vis spectroscopy and FTIR, as greater stability of the inhibitor when encapsulated was observed compared to its free form. However, there was no analysis of the inhibitory activity and encapsulation efficiency to assess whether its action was fully maintained^97^.

In this sense, the studies with TTI have advanced in order to increase the efficiency of the molecule and the stability of the antitryptic activity. Assays were performed to investigate the isolated and conjugated effect of chitosan and whey protein on the incorporation, activity, and stability of TTI. The combination of the encapsulating agents showed excellent incorporation efficiency and TTI stability. In addition, encapsulation in chitosan and whey protein promoted the reduction of the half maximal inhibitory concentration (IC50) and preserved inhibitory activity at temperatures up to 80 °C compared to isolated agents, showing that encapsulation is an interesting strategy to improve the function and stability of TTI^98^.

## Conclusion

Despite the challenges in prospecting for new molecules for the treatment of obesity, the results with trypsin inhibitors are promising. The different physiological effects of these molecules, already attested in the scientific literature, make trypsin inhibitors excellent candidates for action in some chronic diseases involving a network of metabolic variations. However, the mechanisms of action of these molecules is still a field to be explored, as well as its effects on different therapeutic aspects of the disease and its possible side effects. For these reasons, it is recommended to intensify studies and research in this area.
